# Developmental Trajectory of Infant Brain Signal Variability: A Longitudinal Pilot Study

**DOI:** 10.3389/fnins.2018.00566

**Published:** 2018-08-14

**Authors:** Chiaki Hasegawa, Tetsuya Takahashi, Yuko Yoshimura, Sou Nobukawa, Takashi Ikeda, Daisuke N. Saito, Hirokazu Kumazaki, Yoshio Minabe, Mitsuru Kikuchi

**Affiliations:** ^1^Research Center for Child Mental Development, Kanazawa University, Kanazawa, Japan; ^2^Health Administration Center, University of Fukui, Fukui, Japan; ^3^Faculty of Education, Kanazawa University, Kanazawa, Japan; ^4^Department of Computer Science, Chiba Institute of Technology, Narashino, Japan

**Keywords:** infant development, magnetoencephalography (MEG), multiscale entropy, complexity, longitudinal change

## Abstract

The infant brain shows rapid neural network development that considerably influences cognitive and behavioral abilities in later life. Reportedly, this neural development process can be indexed by estimating neural signal complexity. However, the precise developmental trajectory of brain signal complexity during infancy remains elusive. This study was conducted to ascertain the trajectory of magnetoencephalography (MEG) signal complexity from 2 months to 3 years of age in five infants using multiscale entropy (MSE), which captures signal complexity at multiple temporal scales. Analyses revealed scale-dependent developmental trajectories. Specifically, signal complexity predominantly increased from 5 to 15 months of age at higher temporal scales, whereas the complexity at lower temporal scales was constant across age, except in one infant who showed decreased complexity. Despite a small sample size limiting this study’s power, this is the first report of a longitudinal investigation of changes in brain signal complexity during early infancy and is unique in its application of MSE analysis of longitudinal MEG data during infancy. The results of this pilot study may serve to further our understanding of the longitudinal changes in the neural dynamics of the developing infant brain.

## Introduction

Infancy is a period of remarkable neural development in the brain that is reflected by increasing cognitive and behavioral capacities for external circumstances or internal changes in later life (Cao et al., 2017). Recent advances in neuroimaging devices and analysis techniques have been used to visualize the development of brain functions. The human brain is a complex system that is characterized by its astonishing signal variability, which operates over a wide range of temporal and spatial scales. This brain signal variability facilitates learning and optimal environmental adaptation to the changing demands of a dynamic environment (Faisal et al., 2008). This complexity also conveys important information about neural system dynamics and their alterations (reviewed in Stam, 2005; Garrett et al., 2013; Takahashi, 2013).

An entropy-based approach, multiscale entropy (MSE) analysis, has been proposed to estimate the physiological signal complexity on multiple temporal scales using coarse-graining procedures (Costa et al., 2005). This extension to multiple time scales enables the capture of long-range temporal correlations in a time series. MSE has been successfully applied in the investigation of developmental changes in brain signal complexity from infancy through adolescence and into adulthood (McIntosh et al., 2008; Lippe et al., 2009; Polizzotto et al., 2015; Takahashi et al., 2016). However, no study has explored the longitudinal changes in brain signal complexity during the early stages of development despite the significant importance of examining within-subject developmental trajectories (Giedd et al., 1999; Sowell et al., 2004; Shaw et al., 2008). This is due to the large variance in the developmental pattern during infancy (Landa et al., 2012), a period in which developmental disorders frequently emerge (Bolton et al., 2012; Lemcke et al., 2013).

We characterized the trajectory of brain signal complexity of typically developing infants, aged 5 to 36 months, using MSE applied to MEG. MEG is suited for measuring the infant brain because it offers a non-invasive and quiet environment during measurement. Additionally, MEG allows the mother to accompany the infant to provide encouragement and comfort, as well as enabling her to decide whether the experiment should be paused or continued. Furthermore, in the assessment of signal complexity, MEG can directly measure brain magnetic fields in the cortex with high temporal resolution (Kikuchi et al., 2011; Yoshimura et al., 2012; Takahashi et al., 2016).

## Methods

Data for the present study were obtained from an ongoing longitudinal study of infants. In this study, we analyzed five infants (one female and four males) who were 36 months of age at the time of analysis. They were recruited from Kanazawa University at 1 month old, and follow-up examinations and MEG experiments were conducted once a month (ideally every month). Participants had no history of developmental problems at the time of the latest measurement.

All mothers agreed to their infant’s participation in the study and had full knowledge of the experimental nature of the research. Written informed consent was obtained prior to participation. The study was approved by the Ethics Committee of the Kanazawa University Hospital, and all procedures were performed in accordance with the Declaration of Helsinki.

## Experimental Procedure

Magnetoencephalography data were recorded using a 151-channel Superconducting Quantum Interference Device (SQUID) whole-head coaxial gradiometer MEG system for children (PQ 1151 R; Yokogawa/KIT, Kanazawa, Japan) installed at the MEG Center of Ricoh Company, Ltd. (Kanazawa, Japan). During recording, the participant lay supine on a bed in a magnetically shielded room (Daido Steel, Nagoya, Japan) with his or her head inside the MEG system helmet. The infant’s mother and one research member remained in the shielded room to keep the infant comfortable and encourage the infant to maintain a steady body position when necessary. The infants were carefully monitored using a video monitoring system to assess their compliance with the instructions and to record any notable artifacts, such as head motion, inappropriate head position. Before recording, infants or their mother selected a video program according to their preference from a number of video programs (e.g., popular Japanese animations and TV programs). All infants viewed silent video programs projected onto a screen throughout the recording session to promote a consistent state and attention. MEG recordings were conducted every month when possible.

## Data Analysis

Magnetic fields were sampled at 2000 Hz per channel (bandpass filter 0.16–200 Hz). Offline analysis was performed using a BrainVision Analyzer 2 (Brain Products GmbH, Gilching, Germany) and MATLAB (the MathWorks Inc., Natick, MA, United States). The raw MEG data were resampled at 500 Hz with 1.5–60-Hz bandpass and 60-Hz notch filters. MEG data were segmented for 5 s (2500 data points: 5 s × 500 Hz). Artifacts such as eye movements, blinks, cardiac activities, and muscle activities were visually identified and excluded from analyses. The children’s head movements were video monitored throughout the session. At the epoch selection stage, clear head motion artifacts were eliminated by confirmation of head motion in the videos at the time of the MEG artifacts by an MEG expert who was blinded to the identity of the subjects. Contaminated data were also eliminated by an MEG expert who was blinded to the identity of the subjects. A minimum of 50 segments were recorded for each subject. Finally, we randomly selected 50 segments (i.e., a 250 s recording period) from all artifact-free segments of each recording. For each subject, MSE values were calculated separately for each of the selected segments and were then averaged into a single value as the mean MSE.

## MSE Analysis

Multiscale entropy analysis quantifies the complexity of a time series using different time scales (Costa et al., 2002). For the extension to multiple time scales, the original MEG time series {*x*_1_, *x*_2_, …, *x*_N_} is coarse-grained to {*y*_1_(τ), *y*_2_(τ), …, *y*_N/τ_(τ)} by the temporal scale *τ* with non-overlapping windows as follows.

$$\begin{array}{cc} y_{j}(\tau )=(1/\tau )\sum_{i=(j-1)\tau +1}^{j\tau }x_{i}, & 1\leq j\leq N/\tau . \end{array}$$

The complexity of each scale can be measured through the calculation of sample entropy (SampEn), which assesses the predictability of a time series. The SampEn was calculated for each series {*y*_1_(τ), *y*_2_(τ), …, *y*_N/τ_(τ)}. The SampEn is the negative of the logarithmic conditional probability that two sequences of *m* consecutive data points that are mutually similar (within a given tolerance *r*) will remain similar at the next point (*m* + 1) in the dataset (*N*), where *m* is the space of the dimension and *r* is the effective filter for measuring the consistency of a time series (Richman and Moorman, 2000). Considering the MEG time series {*x*_1_, *x*_2_, …, *x*_N_} as observations of a stochastic variable *x*, the dynamic SampEn is defined as

$$h_{\text{sample}}\;(r,\;m,\;N)=-\log_{\text{e}}\;[C_{m+1}\;(r)/C_{m}\;(r)],$$

where *C_m_* (*r*) = {number of pairs (*i*, *j*) with |$z_{i}^{m}$ − $z_{j}^{m}$ | < *r*, *i* ≠ *j*}/{number of all probable pairs, i.e., (*N* − *m* + 1) (*N* − *m*)}. Therein, *z* = *y* (τ); *z^m^* is a vector of an *m* sample time series of (*N* − *m*) length, and |$z_{i}^{m}$ -$z_{j}^{m}$| denotes the distance between points $z_{i}^{m}$ and $z_{j}^{m}$. In this study, we used *m* = 2 and *r* = 0.2. SampEn values were computed for 1–20 scales that correspond to 2–40 ms (Temporal scales in ms = tau ^∗^ 1000 ms/sampling frequency).

## Power Spectral Analysis

Along with MSE calculations, spectral power analysis was performed for each epoch that was used for the MSE calculation as a comparative MSE analysis. We calculated the spectral density (amplitude) using a fast Fourier transform. A Hamming window was applied to each epoch for spectral power analysis.

## Surrogate Analysis

We derived surrogate data using a Fourier transformation to the MEG data to detect non-linearity in the MEG data (Vakorin and McIntosh, 2012; Grandy et al., 2016). Specifically, the time-series of each epoch was Fourier transformed, and then its phase was randomized and applied to an inverse Fourier transform. Using 10 types of seeds for randomization, we derived 10 surrogate data per epoch and then calculated an average value among their SampEn values of surrogate data. We compared the SampEn values for the original time series to the SampEn values for the surrogate data.

## Results

**FIGURE 1:**
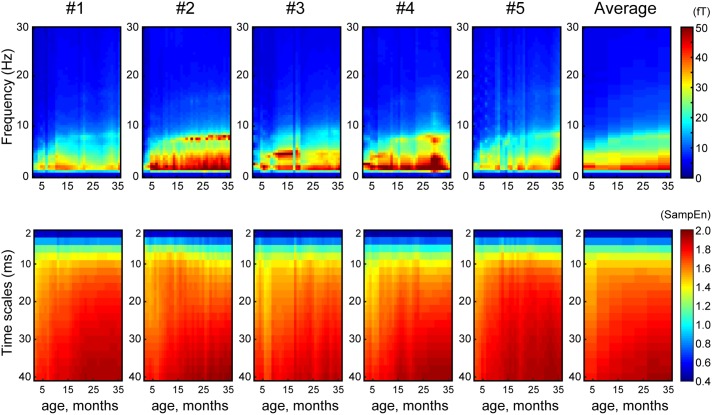
Array plot showing the developmental trajectory of spectral power (top panels) and MSE (bottom panels) for each infant and their average (X-axis, age, months; Y-axis, frequency and time scales).

**FIGURE 2:**
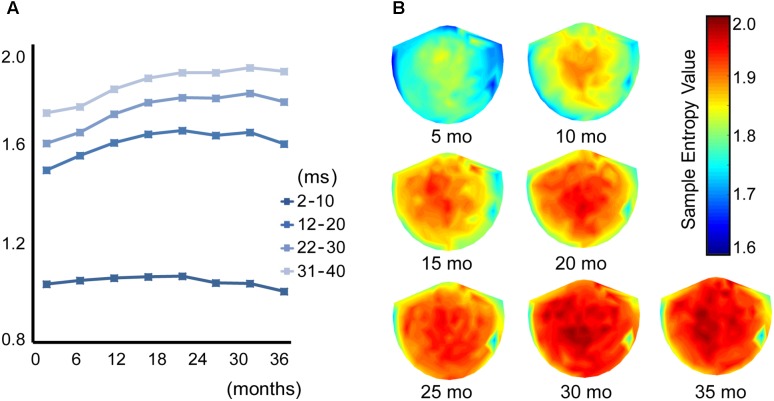
**(A)** Each line shows the trajectory of the averaged MSE value across different time scales in 8 age bins (1–5, 6–10, 11–15, 16–20, 21–25, 26–30, 31–35, and 36–40 months of age). **(B)** Topography of the MSE value at a coarse time scale of 40 ms (scale = 20) across 7 age bins (5, 10, 15, 20, 25, 30, and 35 months of age).

**Figure 1** shows the averaged (across all sensors into a single value) developmental trajectory of the spectral power (**Figure 1**, upper panels) and MSE (**Figure 1**, lower panels) across five infants aged from 5 to 36 months old. All infants demonstrated an increase in the MSE value with age. The Jonckheere-Terpstra test was used to test for an age-related trend in MSE values, and statistically significant age-related trends were identified for coarse time scales (31–40 ms, scales: 16–20) (T_JT_ = 459.0, standard error = 34.8, *z* = 5.6, *p* < 0.001). **Figure 2** shows the averaged developmental trajectory in each time scale bin (**Figure 2A**) and the topography of MSE values across different ages (**Figure 2B**). A more detailed examination of our results revealed that the remarkable increase in MSE identified for longer time scales (31–40 ms, scale: 16–20) was predominantly observed at ages up to 15 months and was found across brain regions (**Figure 2**). After 15 months of age, this increase tended to slow. However, the power spectral analysis also showed an increase in power in the theta and alpha bands. This increase was more prominent after 15 months of age, while the MSE change was more prominent during the earlier infancy periods. Regarding the shorter time scales (2–10 ms, scale: 1–5), the developmental trajectory of MSE varied across subjects. For instance, some infants showed constant MSE values across development, whereas one infant showed a gradual decrease (**Figure 1**, bottom panels).

In the surrogate analysis, we found region- and scale-specific entropy alterations in the surrogate data, which may suggest an inherent non-linearity in the MEG data (data not shown). Specifically, in the surrogate data, the SampEn increased near the frontal and temporo-occipital regions. Interestingly, this region-specific SampEn alteration was more prominent for smaller temporal scales (less than 20 ms) and was frequently identified during early infancy (5–10 months of age).

## Discussion

The neurodevelopmental trajectory of infancy has received much attention because infancy is a critical period of brain development in which cognitive and behavioral abilities are enhanced (Cao et al., 2017) and neurodevelopmental disorders, such as autism spectrum disorder (ASD), are predicted to develop. This is the first longitudinal investigation of how brain signal complexity, which represents neural system dynamics, changes during infancy. The analysis revealed scale-dependent developmental trajectories of MEG signal complexity. Specifically, we found an increase in signal complexity for longer time scales, whereas the changes in complexity varied across infants for shorter time scales.

Many studies have investigated age-related signal complexity changes from late childhood into adulthood. Polizzotto et al. (2015) examined the MSE of resting-state EEG results in healthy subjects aged 8–22 years old. They reported an age-related increase in entropy in lower scales and a decrease in entropy for higher scales. McIntosh et al. (2008) calculated MSE changes in EEG during a face recognition visual memory task in children (8–15 years old) and young adults (20–33 years old). They found an age-related increase in EEG complexity that was significantly correlated with the accuracy of task performance. This observation was replicated by the same group using MEG (Misic et al., 2010), confirming the characteristic shape of the MSE curve and its prominent task-dependent increase during development. We have also demonstrated an age-related increase in MEG signal complexity. However, enhanced complexity was identified in children with ASD, particularly in earlier childhood (Takahashi et al., 2016). Compared to the changes that occur during the period from childhood to adolescence, brain signal complexity during infancy has been addressed by few studies. Lippe et al. (2009) investigated EEG signal complexity in response to visual and auditory stimulation in children ranging from 1 month to 5 years of age. They found a task-dependent increase in EEG complexity with aging. However, these studies were based on a cross-sectional study design. Despite the small number of subjects, a unique aspect of this study is that we longitudinally investigated the development of MEG complexity during infancy.

In these contexts, our study provides the longitudinal underpinnings for the concept of significant shifts in brain signal complexity with aging (Garrett et al., 2013). Notably, for higher scales, we captured a robust developmental MSE profile across infants and across MEG sessions despite conditional inconsistencies (i.e., selected videos, emotions, or physical conditions), which may indicate the potential usefulness of MSE as a reliable and clinically useful trait biomarker of the infant brain. For instance, we have demonstrated a linear age-related increase in complexity at higher scales across 40–110 month-old children (Takahashi et al., 2016). Additionally, enhanced brain signal variability was observed in children with ASD, which was conformed for younger children. On the other hand, Bosl et al. (2011) examined resting-state EEG complexity by MSE in typically developing infants and infants with a high risk of ASD across the ages of 6–24 months, and they found consistently lower EEG complexity at higher scales in the high-risk group, particularly at 9–12 months of age. These inconsistent results may be attributed to the different age ranges of these two previous studies on children with ASD.

Considering biological background, the observed rapid increase in MEG complexity in the high time scale (i.e., lower frequency range) at approximately 5–15 months old might demonstrate the development of long-range network-related cognitive processing. Given that long-range communication between multiple brain areas is driven by slow waves (i.e., theta and beta waves) (Wang, 2010), MEG complexity in a high time scale (i.e., lower frequency range) may be useful and a non-invasive biomarker of brain maturation in infants. Pujol et al. (2006) assessed myelination from birth to 3 years of age in children’s brains using three-dimensional MRI imaging. Intriguingly, this volumetric study demonstrated that a period of rapid myelination started after the 5th month and reached the mature appearance by the 18th month, and the study revealed the relationship with vocabulary acquisition in children. This period of rapid myelination is almost the same as the period in which we observed a rapid change in the present study.

However, contrary to the developmental trajectory for higher scales, the developmental trajectory of MSE for lower scales is diverse across infants, and the reason for this difference remains unclear. Lippe et al. (2009) reported a rapid increase in complexity at lower scales, especially during the early stage of infancy (1–2 months old vs. 2–8 months old) that is followed by a gradual increase. This may suggest the possibility that complexity at lower scales (corresponding to ≤16 ms) saturates by 8 months of age. This may partially explain our finding of a constant complexity value across age after 5 months of age at lower scales. Theoretically, SampEn at finer (i.e., lower) time scales is based on wider frequencies, whereas coarser (i.e., higher) time scales are based on narrower frequencies (i.e., high frequency is filtered out). Signal variabilities in different frequencies must be reflected by differences in time scale. Therefore, a frequency-specific role in the differentiation of cognitive processing (Fries, 2015) and differences in maturational speed (Uhlhaas et al., 2009) may underlie these contradictory findings between the results from high and low time scales.

Surrogate analysis showed a region- and scale-specific increase in surrogate data compared to that in MSE from original data, which may suggest an inherent non-linearity in the MEG data. Furthermore, the developmental trajectory of the spectral power and MSE differed. Specifically, an increase in the power spectral seemed to be prominent after 12 months of age, whereas an increase in MSE emerged from early infancy until 15 months of age. Therefore, we assume that the enhancement in MSE with development may be associated with non-linear processes and may be independent of spectral power. In addition, as the outputs of neuronal networks are produced by interactions due to both local dense interconnectivity and sparse long-range excitatory projections (Friston et al., 1995), the resulting dynamics could be expected to operate at multiple scales.

Some potential limitations of the present study must be considered. First, despite frequent MEG recording, we were only able to follow five infants, which precluded statistical evaluation. Second, we did not correct for cognitive behavioral or psychological assessments, which might strengthen our claims. Third, the confounding influence of head motion cannot be excluded from potentially influencing the MSE results. Finally, as a technical consideration, the recent advent of cortical source localization techniques was not applied due to difficulties in performing MRI on infants. Although several limitations must be considered, our findings for the examination of MEG signal variability using MSE may add another dimension to the previously identified neural dynamics of development and may provide useful biomarkers for typically and abnormally developing brains.

## Author Contributions

YY, YM, and MK designed the study. CH, YY, and HK recruited the participants. CH, YY, TI, and DS performed the experiments. CH, SN, and TT analyzed the results and wrote the manuscript. All authors participated in revising the manuscript and approved the final draft of the manuscript.

## Conflict of Interest Statement

The authors declare that the research was conducted in the absence of any commercial or financial relationships that could be construed as a potential conflict of interest. The reviewer C-KP and the handling Editor declared their shared affiliation.

## Abbreviations

MEG: magnetoencephalography.
